# Elective caesarean section on maternal request prior to 39 gestational weeks and childhood psychopathology: a birth cohort study in China

**DOI:** 10.1186/s12888-019-2012-z

**Published:** 2019-01-14

**Authors:** Kun Huang, Shuangqin Yan, Xiaoyan Wu, Peng Zhu, Fangbiao Tao

**Affiliations:** 1 School of Public Health, Anhui Provincial Key Laboratory of Population Health and Aristogenics, Anhui Medical University, No 81 Meishan Road, Hefei, Anhui Province China; 2 Ma’anshan Maternal and Child Health Center, No 72 Jiashan Road, Ma’anshan, Anhui Province China

**Keywords:** Caesarean section on maternal request, Early term, Preterm, Emotion and behavior, Preschool children

## Abstract

**Background:**

The recommendation of non-indicated caesarean section (CS) after 39 gestational weeks has been announced based on evidence of maternal and infant physiological effects. The potential psychological risks have not been acknowledged. This study aims to investigate emotional and behavioral problems in pre-school children born with elective CS (ECS) on maternal request prior to 39 weeks.

**Methods:**

Pregnant women within 12 gestational weeks between November 2008 and October 2010 were invited to participate in the China-Anhui Birth Cohort Study (C-ABCS). They were asked to complete a self-administered questionnaire respectively in 1st and 3rd trimester of pregnancy to collect basic maternal characteristics. Pregnant complications and delivery modes were abstracted from medical notes. Their singleton live births were followed up at preschool age. Strengths and Difficulties Questionnaires (SDQ) were completed by parents to assess children’s emotional and behavioral problems. A total of 3319 mother-child pairs were put into the final analysis. Descriptive analysis and binary logistic regression analysis were used to assess the impact of delivery modes on abnormalities in SDQ dimensions at various gestational ages.

**Results:**

The prevalence of ECS on maternal request prior to 39 weeks, at 39–40 weeks, and after 41 weeks was 16.6, 23.7 and 15.9%, respectively. Compared with those born vaginally, children born with ECS on maternal request were more likely to have total difficult problems (RR 1.519, 95% confidence interval 1.077 to 2.142). ECS on maternal request was the independent predictor of emotional problems (3.479, 1.676 to 7.222) and total difficult problems (2.172, 1.175 to 4.016) in children born prior to 39 gestational weeks.

**Conclusion:**

Children delivered by ECS on maternal request have an increased risk to have emotional and behavioral problems prior to 39 gestational weeks at preschool age. The potential psychological implication prior to 39 weeks has been added to the roster of impacts of ECS on maternal request. Further research is needed to probe the potential biological mechanisms.

**Electronic supplementary material:**

The online version of this article (10.1186/s12888-019-2012-z) contains supplementary material, which is available to authorized users.

## Introduction

Caesarean section (CS) rate is rising dramatically around the world [1]. WHO Global Survey of Maternal and Perinatal Health (WHOGS; 2004–08) and WHO Multi-Country Survey of Maternal and Newborn Health (WHOMCS; 2010–11) have shown that the CS rate increased overall between the two surveys (from 26·4% in the WHOGS to 31·2% in the WHOMCS) in all countries except Japan. The rate is highest in China between the two surveys and has increased from 46.2 to 47.6% [2]. Elective CS (ECS) scheduled before the labor is a major contributor to this uprising trend [3]. The rate of CS on maternal request (non-indicated CS), a subgroup of ECS, is high and growing. It has accounted for 4–18% of CS births in USA and for 36% in south-east China and thus becomes an emerging global public health concern [4, 5].

Childhood psychopathology related to mode of delivery has drawn wide attention recently. Emotional and behavioral problems are areas that require more concern because they are potentially modifiable and are related with later-life social development [6]. Researchers have studied the effect of delivery mode on children’s development in these areas, but the conclusions are controversial. Some studies have observed developmental delay in personal social and gross motor domains [7] and more emotional disturbances among children born from maternally-requested ECS [8]. While others reported no association [9] or even an association between maternally-requested ECS and a lower risk of childhood psychopathological problems [3].

Simultaneously, mean gestational age has decreased globally [10]. This trend is primarily owing to the rise in ECS [11]. The absolute increase of 8.9% of early term infants (defined as 37–38 gestational weeks) during the past decade in USA is largely due to an increase in CS on request [12]. A regional audit has shown that 44% of ECS was carried out before 39 weeks, maternal request being one of the top three reasons for ECS [13]. Not just preterm birth will cause adverse consequences [14, 15], early term birth, which was ever considered as term birth, is also recognized to have potentially detrimental health effect, such as increased mortality and hospital stay, greater complications in subsequent pregnancies, short-term respiratory morbidities and newborn intensive care admissionsin in elective early term births [16–22]. Researchers also found a dose-dependent relationship of special education needs (SEN) with gestation. Communication impairments and lower general cognitive ability were more common in infants born early term and accounts for more cases of SEN [14, 15].

Given the potential risks in CS on maternal request, educational and policy initiatives have been led to eliminate the non-indicated CS prior to 39 gestational weeks by American College of Obstetricians and Gynecologists and National Institute of Clinical Excellence [23, 24]. Nevertheless, whether the recommendation can be universally applied is questionable as it is much based on physiological evidence or expert opinion [25]. Neither group has acknowledged the children’s potential psychological risk. Meanwhile, the gestational age of fetal maturity may vary by race [26], and there were notable racial disparities in race in serious neonatal morbidities in preterm infants [27]. Previous researches on offspring’s emotional and behavioral development have simply concerned delivery mode and mostly by retrospective design [3, 7, 8], or solely looked into the gestational age and highly inclined to preterm births [28, 29]. The population-based prospective studies on the combinative effects of delivery mode and gestational length on psychological outcomes are scarce. In the present study, based on a Chinese birth cohort, we recruited women with singleton live births, investigated emotional and behavioral development in pre-school children born with non-indicated ECS at different gestational ages.

## Methods

### Participants

Participants of this study came from the China-Anhui Birth Cohort (C-ABCS). This cohort was set up between November 2008 and October 2010 in six cities in Anhui province, China. Anhui province is located in the southeast of China. In 2017, it has a resident population of 62.5 million, 52.0% of which live in urban areas. GDP per capita is 44, 206 yuan (us $6547). The cohort profile was published elsewhere [30].

**Fig. 1:**
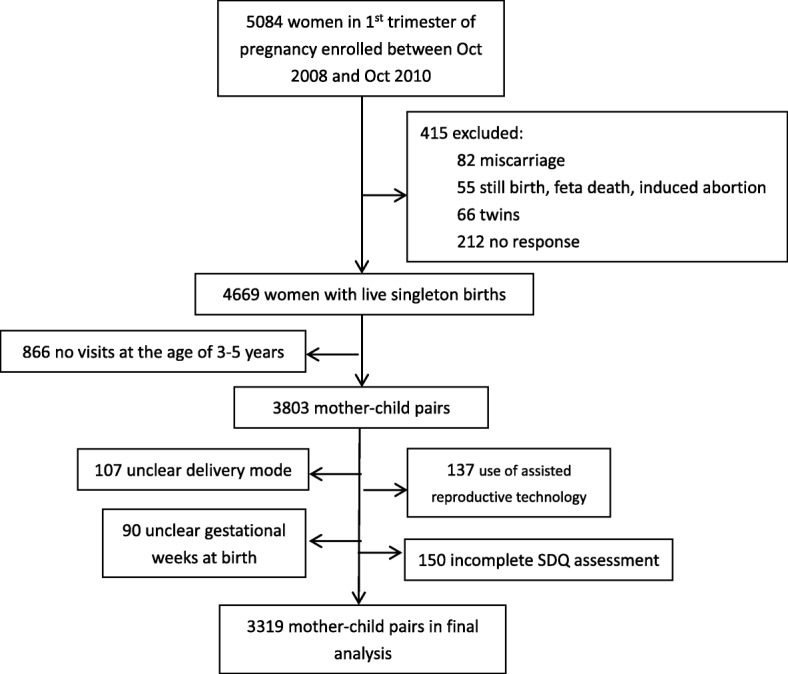
Flow chart of participants recruited in this study

Mother-child pairs in this article were from one of the six cities, Ma’anshan city. The city was selected because of a stable population, having a high-quality child health care system and a strong research team in children’s development. The enrolling criteria of participants included: 1) within 12 gestational weeks who came to maternal and child health center for the first antenatal visit; 2) lived in Ma’anshan city for over 6 months; 3) willing to have childbirth in the center; 4) could understand the questionnaire and correctly answer the questions; 5) without mental diseases. A total of 5084 women were recruited in original cohort and 3319 mother-child pairs with full data were put into the final analysis. Figure 1.

### Study procedures

Women were asked to complete a self-administered questionnaire respectively in the 1st and 3rd trimester of pregnancy. Children were followed up when they were at the age of 3–5 years. Their parents were invited to kindergartens and fill in questionnaires (Additional file 1).

#### Maternal characteristics

During the 1st visit of pregnancy, maternal demographic information was collected. It included maternal age, maternal educational years (total years that women had been educated for), residence and family income. Previous adverse pregnant outcomes were reported by women, including spontaneous abortion, induced abortion, preterm, difficult vaginal delivery, fetal death or stillbirth, newborn death, ectopic pregnancy, cystic mole, malformation. Women who had any one of above-mentioned outcomes were defined to have previous adverse pregnant outcome. Women who had no previous experience of childbirth, regardless of gestational age, delivery mode or newborn’s health status, were defined as primipara. Usage of assisted reproductive technology for the index pregnancy was reported by answering yes or no. Maternal weight and height were measured by research team members. The weight was regarded as the maternal pre-pregnancy weight. Pre-pregnancy body mass index (BMI) was then calculated dividing pre-pregnancy weight (kg) by body height (m)^2^. Based on the guideline of Coorperative Meta-analysis Group of Working Group on Obesity in China (WGOC), we defined the level of BMI < 18.5 for underweight,24 to 27.9 for overweight and ≥ 28 for obesity for Chinese adults [31].

Pregnancy-related anxiety was assessed in 1st and 3rd trimester of pregnancy by pregnancy-related anxiety questionnaire developed by research team. The coefficient of retest reliability and Cronbach α coefficient were 0.786 and 0.811, respectively [32]. The final questionnaire consisted of 13 items covering anxiety regarding women’s own health, fetal health and childbirth. Women were asked to self-rate their perception by strength varying from no worries, occasionally worried, often worried to always worried. Scores ranged from 13 to 52, higher scores indicating higher level of pregnancy-related anxiety. Women whose total scores over 24 were classified as having pregnancy-related anxiety [33].

Pregnant complications and delivery modes were abstracted from medical notes. Main complications covered gestational diabetes, hypertensive disorders in pregnancy and intrahepatic cholestasis of pregnancy. Women who had any one of these diseases were considered as having pregnant complications. As to the delivery mode, due to the very small sample size, instrumental delivery and assisted breech delivery were excluded from data analysis. Final delivery mode was classified as vaginal delivery, elective caesarean section (ECS) and emergency CS. ECS was further grouped into ECS with medical indications and ECS on maternal request in the absence of any medical indications.

#### Children’s characteristics

Children’s gender, birth weight, Apgar scores, gestational ages at birth were from medical notes. Infants born less than 36 6/7 weeks were defined as preterm, 37 0/7 weeks through 38 6/7 weeks were early term. These two groups were merged as prior to 39 weeks. 39 0/7 weeks through 40 6/7 weeks were full term and 41 0/7 weeks and beyond were late/post term [25]. Feeding pattern during 4–6 postnatal months was reported by parents.

Strengths and Difficulties Questionnaire (SDQ) was completed by parents to identify children’s emotional and behavioral problems. It consisted of 25 items rated on a 3-point Likert scale: 1 “not true”, 2 “somewhat true” and 3 “certainly true”. These 25 items were divided into five subscales named as emotional symptoms, conduct problems, hyperactivity, peer problems and pro-social behaviors. Higher scores in the former 4 subscales indicated greater symptom severity, and they were summed up to generate a total difficulties score. The subscale of pro-social behaviors was scored positively and high scores indicated the presence of more adaptive behaviors. Normative data for Chinese urban population with bandings and cut-offs for borderline and abnormal scores were described by Shanghai Mental Health Centre, China [34]. The following cut-off points were used to identify abnormal emotional and behavioral development: emotional symptoms (≥5), conduct problems (≥4), hyperactivity (≥8), peer problems (≥6), total difficulties (≥17), and pro-social behaviors (≤4). The internal consistency, test retest reliability, convergent validity and discriminant validity were acceptable as described by Du Y et al. [34].

### Data analysis

Data analysis was performed using the Statistical Package of Social Sciences and Problem Solutions (SPSS, version 16.0).

Maternal and children’s characteristics among four types of delivery modes were described as means, standard deviations for continuous variables, and as frequencies for categorical indicators. Analysis of variance (one-way ANOVA, with LSD’s multiple comparison tests) and χ^2^ tests were adopted to compare the differences among continuous variables and categorical indicators, respectively. In each gestational group, prevalence of abnormalities in SDQ dimensions was compared by χ^2^ tests by delivery modes. When multiple comparison tests were carried out for categorical indicators, number of analyses was counted and threshold for significance was adjusted based on number of tests completed. Binary logistic regression analysis was used to assess the impact of delivery modes on abnormalities in SDQ dimensions at various gestational ages. Maternal age, maternal educational level, residence, family income, previous adverse pregnant outcomes, pre-pregnancy BMI, parity, pregnant complications, pregnancy-related anxiety, children’s gender, birth weight and 5 min Apgar scores were adjusted in the models. In addition, sensitive analysis was conducted in this study. Firstly, we compared the basic maternal characteristics of those who were recruited in final data analysis with those who dropped out due to incomplete data to assess potential selection bias by independent *t* tests and χ^2^ tests. Secondly, we had run the model restricting to boys. In addition, pregnancy-related anxiety [35–37], pregnant complications and pre-pregnancy BMI [37–39] were key determinants on children’s psychological development. Being adjusted for the factors in logistic regression models may not fully eliminate their effects on children’s emotional and behavioral development. Thus we excluded women with pregnant complications, pregnancy-related anxiety, abnormal pre-pregnancy BMI and repeatedly run the model. In the third, as most emergency caesarean sections were usually performed in pregnancies in which a vaginal delivery was planned initially but an indication for caesarean section has developed. Risks of complications from the emergency operation may be increased. Here we had put emergency caesarean section as the reference group, re-run the model and compared the prevalence of abnormities in SDQ between children born via ECS and those born via emergency CS. Statistical significance was set at the alpha = 0.05 level.

## Results

### Prevalence of caesarean section on maternal request in different gestational ages

Among 3319 children, 920 (27.7%) were born prior to 39 gestational weeks, including 116 (3.5%) preterm and 804 (24.2%) early term children. As to the delivery mode, 2171 (65.4%) children were born with CS, and 693 (20.9%) were ECS on maternal request. As shown in Table 1, the rate of ECS on maternal request in children born prior to 39 weeks, 39-40 weeks and ≥ 41 weeks was 16.6, 23.7 and 15.9%, respectively.

**Table 1:** Distribution of delivery modes in various gestational ages among children

Children with various gestational weeks	Delivery mode			
	Vaginal delivery (*n* = 1148)	ECS with medical indications (*n* = 1154)	ECS on maternal request (*n* = 693)	Emergency caesarean section (*n* = 324)
< 39 weeks (*n* = 920)	307/33.4	392/42.6	153/16.6	68/7.4
39–40 weeks (*n* = 2040)	720/35.3	652/32.0	483/23.7	185/9.1
≥41 weeks (*n* = 359)	121/33.7	110/30.6	57/15.9	71/19.8

### Baseline maternal and children characteristics in relation to delivery mode

Women with non-indicated ECS were younger than those with vaginal delivery (P<0.001) and older than those with emergency CS (*P* = 0.006). The score of pregnancy-related anxiety in 3rd trimester of pregnancy was significantly higher in women taking CS on request than women with vaginal delivery (*P* = 0.001) and women having ECS with medical indications (*P* = 0.001). Women who chose non-indicated ECS were more likely to have previous adverse pregnant outcomes (*P* = 0.001) and pregnancy complications (*P* = 0.005) than those who chose vaginal delivery.

Women who had ECS with medical indications were more educational than those with vaginal delivery (*P* = 0.004) and with ECS on maternal request (*P* = 0.045), and had significantly higher pre-pregnancy BMI than those with vaginal delivery (*P*<0.001), with non-indicated ECS (*P*<0.001) and with emergency CS (*P* = 0.013). Babies born by vaginal deliveries had lowest birth weights (*P*<0.001) and those born by emergency CS were the heaviest (*P*<0.05) (Table 2).

**Table 2:** Baseline maternal and children’s characteristics in relation to mode of delivery

Characteristics	Vaginal delivery (*n* = 1148)	ECS with medical indications (*n* = 1154)	ECS on maternal request (*n* = 693)	Emergency caesarean section (*n* = 324)	Total
*Maternal characteristics*					
Maternal age (years) (mean ± SD) **	26.1 ± 3.1	27.3 ± 3.6	27.1 ± 3.5	26.5 ± 2.9	26.8 ± 3.4
Maternal educational years (mean ± SD)*	12.9 ± 3.6	13.3 ± 3.2	12.9 ± 3.2	13.3 ± 3.1	13.0 ± 3.3
Residence (n/%)**^a^					
Urban areas	981/85.7	1041/90.3	614/88.6	271/83.9	2907/87.7
Rural areas	164/14.3	112/9.7	79/11.4	52/16.1	407/12.3
Family monthly income per capita (n/%)**^b^					
≤ 2000 RMB yuan	696/60.8	640/55.5	409/59.0	177/54.8	1922/58.0
2001–7999 RMB yuan	439/38.4	509/44.1	273/39.4	145/44.9	1366/41.2
≥ 8000 RMB yuan	9/0.8	4/0.3	11/1.6	1/0.3	25/0.8
Previous adverse pregnant outcomes (n/%) **	500/43.6	585/50.7	371/53.5	140/43.2	1596/48.1
Pre-pregnancy BMI (kg/m^2^) (mean ± SD)**	19.9 ± 2.1	20.6 ± 2.7	20.1 ± 2.3	20.2 ± 2.1	20.2 ± 2.3
Primiparity (n/%)	1094/95.3	1087/94.2	660/95.2	315/97.2	3156/95.1
Pregnant complications (n/%)*	147/12.8	196/17.0	110/15.9	41/12.7	494/14.9
Pregnancy-related anxiety scores in 1st trimester of pregnancy (mean ± SD)	20.7 ± 4.9	21.0 ± 5.0	21.2 ± 5.0	20.6 ± 4.8	20.9 ± 4.9
Pregnancy-related anxiety in 1st trimester of pregnancy (n/%)	270/23.5	286/24.8	190/27.4	79/24.4	825/24.9
Pregnancy-related anxiety scores in 3rd trimester of pregnancy (mean ± SD)**	19.2 ± 4.1	19.2 ± 4.3	19.9 ± 4.5	19.6 ± 4.5	19.4 ± 4.3
Pregnancy-related anxiety in 3rd trimester of pregnancy (n/%)	152/13.2	144/12.5	110/15.9	54/16.7	460/13.9
*Children’s characteristics*					
Children’s age (years) (mean ± SD)**	3.8 ± 0.5	3.8 ± 0.5	3.8 ± 0.6	3.7 ± 0.5	3.8 ± 0.5
Children’ gender (n/%)					
Male	604/52.6	643/55.7	352/50.8	176/54.3	1775/53.5
Female	544/47.4	511/44.3	341/49.2	148/45.7	1544/46.5
Birth weight (g) (mean ± SD) **	3320.2 ± 402.5	3472.5 ± 492.3	3450.5 ± 380.4	3535.3 ± 396.6	3421.4 ± 437.7
A pgar score					
1 min	9.9 ± 0.6	9.9 ± 0.6	10.0 ± 0.5	10.0 ± 0.7	9.9 ± 0.6
5 min	10.0 ± 0.3	10.0 ± 0.3	10.0 ± 0.3	10.0 ± 0.4	10.0 ± 0.3
Full breastfeeding during 4–6 postnatal months (n/%)	249/21.7	246/21.3	144/20.8	74/22.8	713/21.5

* *P*<0.05, ** *P*<0.01

^a^3 missing values in women with vaginal delivery, 1 missing value in women having ECS with medical indications and 1 missing value in women with emergency caesarean section

^b^4 missing values in women with vaginal delivery, 1 missing value in women having ECS with medical indications and 1 missing value in women with emergency caesarean section

### Abnormities in SDQ by delivery mode in children with various gestational ages

When children were classified by certain threshold in each dimension, it was found that in children born prior to 39 gestational weeks, there were significant differences in the prevalence of emotional problems, conduct problems and total difficult problems. All the three rates of abnormities were highest in children born with ECS on maternal request, being 14.4, 13.1 and 15.0%, respectively. Multiple comparisons were performed with the threshold for significance = 0.008. It revealed that in terms of emotional problems, children born with ECS on maternal request had significantly higher rates than children with vaginal delivery (*P*<0.001), children with indicated ECS (*P*<0.001) and those with emergency CS (*P* = 0.004). As to conduct problems, the prevalence was significantly higher in children with ECS on maternal request than those with indicated ECS (*P* = 0.005) (Table 3).

**Table 3:** Abnormities in SDQ by delivery mode in children with various gestational ages (n/%)

Children with various gestational weeks	SDQ dimensions	Delivery mode				Total
		Vaginal delivery	ECS with medical indications	ECS on maternal request	Emergency caesarean section	
< 39 weeks	Emotional symptoms^**^	13/4.2	21/5.4	22/14.4	1/1.5	57/6.2
	Conduct problems^*^	24/7.8	23/5.9	20/13.1	3/4.4	70/7.6
	Hyperactivity	31/10.1	33/8.4	15/9.8	4/5.9	83/9.0
	Peer problems	14/4.6	5/1.3	5/3.3	3/4.4	27/2.9
	Total difficult problems^*^	23/7.5	31/7.9	23/15.0	3/4.4	80/8.7
	Pro-social behaviors	30/9.8	40/10.2	17/11.1	5/7.4	92/10.0
39–40 weeks	Emotional symptoms	45/6.2	45/6.9	28/5.8	13/7.0	131/6.4
	Conduct problems	52/7.2	38/5.8	44/9.1	8/4.3	142/7.0
	Hyperactivity	49/6.8	39/6.0	38/7.9	6/3.2	132/6.5
	Peer problems	18/2.5	19/2.9	16/3.3	4/2.2	57/2.8
	Total difficult problems	48/6.7	40/6.1	45/9.3	11/5.9	144/7.1
	Pro-social behaviors	83/11.5	64/9.8	62/12.8	16/8.6	225/11.0
≥41 weeks	Emotional symptoms	7/5.8	5/4.5	1/1.8	8/11.3	21/5.8
	Conduct problems	11/9.1	15/13.6	3/5.3	7/9.9	36/10.0
	Hyperactivity	17/14.0	7/6.4	4/7.0	9/12.7	37/10.3
	Peer problems	3/2.5	2/1.8	1/1.8	3/4.2	9/2.5
	Total difficult problems	10/8.3	6/5.5	2/3.5	11/15.5	29/8.1
	Pro-social behaviors	11/9.1	10/9.91	8/14.0	7/9.9	36/10.0
Total	Emotional symptoms	65/5.7	71/6.2	51/7.4	22/6.8	209/6.3
	Conduct problems	87/7.6	76/6.6	67/9.7	18/5.6	248/7.5
	Hyperactivity	97/8.4	79/6.8	57/8.2	19/5.9	252/7.6
	Peer problems	35/3.0	26/2.3	22/3.2	10/3.1	93/2.8
	Total difficult problems^*^	81/7.1	77/6.7	70/10.1	25/7.7	253/7.6
	Pro-social behaviors	124/10.8	114/9.9	87/12.6	28/8.6	353/10.6

^*^P < 0.05, ^**^P < 0.01

When the group prior to 39 weeks was divided into preterm and early term, we found a more possibility of conduct problems (42.9%) and total difficult problems (42.9%) in preterm children born with ECS on maternal request compared with other three delivery modes. The rate of emotional symptoms in early-term children born with vaginal delivery, with indicated ECS with ECS on maternal request and with emergency CS was 3.9, 5.0, 13.7 and 1.6%, respectively. The difference was statistically significant (*P* < 0.01) (data not shown). There were no significant differences in SDQ dimensions, however, among various delivery modes in children born at 39–40 weeks and beyond 41 weeks.

As a whole, children born by ECS on maternal request had highest prevalence in total difficult problems (10.1%) irrespective of gestational weeks. The difference was significant between children with ECS on maternal request and children with indicated ECS (*P* = 0.007) (Table 3).

### Effect of ECS on maternal request on SDQ in children born with various gestational ages

In children prior to 39 weeks, compared with vaginal delivery, ECS on maternal request was significantly associated with emotional problems (RR 3.479, 95% confidence interval 1.676 to 7.222) after adjusting for potential maternal and infant characteristics. ECS with medical indications decreased the risk of peer problems (0.247, 0.087 to 0.701). Non-indicated ECS was also independent predictive factor of total difficult problems (2.172, 1.175 to 4.016). No significant effect was observed of ECS on maternal request on SDQ dimensions among children born at 39–40 weeks and beyond 41 weeks. Overall, despite of gestational ages, children born with ECS on maternal request had more possibilities to have total difficult problems compared with those born with vaginal delivery (1.519, 1.077 to 2.142 (Table 4).

**Table 4:** Binary logistic regression analysis on effect of delivery mode on SDQ in children born with various gestational ages [RR (95%CI)]

SDQ dimensions	Mode of delivery	< 39 weeks	39–40 weeks	≥41 weeks	Total
Emotional problems	ECS with medical indications	1.274(0.619–2.620)	1.127(0.730–1.739)	0.782(0.235–2.598)	1.079(0.759–1.532)
	ECS on maternal request	3.479(1.676–7.222)	0.871(0.529–1.434)	0.298(0.035–2.528)	1.243(0.845–1.826)
	Emergency caesarean section	0.373(0.048–2.918)	1.059(0.544–2.062)	2.193(0.733–6.561)	1.200(0.719–2.004)
Conduct problems	ECS with medical indications	0.737(0.403–1.348)	0.794(0.512–1.230)	1.380(0.576–3.306)	0.913(0.658–1.265)
	ECS on maternal request	1.746(0.925–3.297)	1.254(0.820–1.918)	0.560(0.145–2.155)	1.314(0.937–1.842)
	Emergency caesarean section	0.563(0.163–1.944)	0.577(0.269–1.241)	0.990(0.352–2.786)	0.759(0.448–1.284)
Hyperactivity	ECS with medical indications	0.791(0.464–1.349)	0.863(0.554–1.344)	0.376(0.148–0.956)	0.823(0.600–1.130)
	ECS on maternal request	0.959(0.494–1.859)	1.126(0.721–1.757)	0.411(0.130–1.301)	0.965(0.681–1.367)
	Emergency caesarean section	0.569(0.191–1.695)	0.470(0.197–1.120)	0.696(0.280–1.733)	0.643(0.380–1.086)
Peer problems	ECS with medical indications	0.247(0.087–0.701)	1.106(0.573–2.134)	0.716(0.117–4.368)	0.704(0.420–1.179)
	ECS on maternal request	0.705(0.248–2.008)	1.270(0.638–2.526)	0.703(0.071–6.913)	1.033(0.599–1.780)
	Emergency caesarean section	1.087(0.300–3.933)	0.847(0.283–2.542)	1.731(0.340–8.822)	1.029(0.502–2.108)
Total difficult problems	ECS with medical indications	0.953(0.537–1.692)	0.982(0.627–1.536)	0.629(0.221–1.792)	1.004(0.720–1.399)
	ECS on maternal request	2.172(1.175–4.016)	1.440(0.929–2.233)	0.404(0.085–1.907)	1.519(1.077–2.142)
	Emergency caesarean section	0.576(0.168–1.977)	0.881(0.443–1.750)	1.817(0.716–4.611)	1.086(0.672–1.755)
Pro-social behaviors	ECS with medical indications	1.058(0.635–1.763)	0.817(0.578–1.155)	1.125(0.440–2.879)	0.894(0.682–1.173)
	ECS on maternal request	1.221(0.645–2.312)	1.120(0.788–1.594)	1.454(0.508–4.160)	1.191(0.887–1.599)
	Emergency caesarean section	0.711(0.263–1.923)	0.722(0.411–1.267)	1.070(0.375–3.053)	0.767(0.498–1.181)

Vaginal delivery as the reference group

Adjusted for maternal age, maternal education level, residence, family income, previous adverse pregnant outcomes, pre-pregnancy BMI, pregnant complications, parity, pregnancy-related anxiety, children’ gender, birth weight and 5 min Apgar score

In sensitive analysis, more women came from urban areas in those who dropped out of the analysis. More proportion of women had higher monthly income per capita (≥8000 RMB yuan) in the recruited group. There were no differences in previous adverse pregnant outcomes, pre-pregnancy BMI, parity or pregnancy-related anxiety between the two groups (Additional file 2: Table S1). When we restricted the participants to boys and re-run the model, the results did not change materially (Additional file 2: Table S2). Similarly, exclusion of women with pregnancy-related anxiety, pregnant complications, or abnormal pre-pregnancy BMI did not change the findings materially (Additional file 2: Table S3-S5).

When using emergency CS as reference, in children born prior to 39 gestational weeks, ECS on maternal request increased the risk of emotional problems (9.326, 1.220 to 71.291) and total difficult problems (3.687, 1.059 to 12.835). ECS with medical indications, however, decreased the possibility of children’s peer problems (0.227, 0.052 to 0.997) (Additional file 2: Table S6).

## Discussion

In this prospective birth cohort study in China, the overall CS rate had reached 65.4%, and the rate of ECS on maternal request was 20.9%. The prevalence of ECS on maternal request prior to 39 weeks, at 39–40 weeks and beyond 41 weeks was 16.6, 23.7 and 15.9%, respectively. ECS on maternal request was the independent predictor of emotional problems and total difficult problems in children born prior to 39 gestational weeks at preschool age.

Although WHO has stated that no robust evidence existed for ideal CS rate, the rate observed in our study was very high. It was higher than that reported by WHO surveys [2], and that observed in Shanghai (38.8%, 2007) and Wenzhou (50.3%, 2008) in China [40, 41]. The rate of non-indicated ECS was lower than that in Shanghai (24.7%, 2007) while higher than that in Wenzhou (18.25%, 2008) [40, 41].

It is reported that at population level, CS rates over 10% are not related with improvements in mothers and newborns [42]. Previous studies failed to find significant associations between delivery mode and infant and childhood psychopathology [43]. Even the least prevalence of emotional or behavioral problems in children born by ECS was reported [3]. In our study, we observed a higher possibility of total difficult problems in children born by ECS on maternal request. When gestational age was concerned, the associations were centrally distributed in the group prior to 39 weeks. Most notably, higher prevalence of emotional problems in children born early term by ECS on maternal request was observed at the first time in our study. Interestingly, when emotional problems were controlled in regression model, the RR (95%CI) of total difficult problems for ECS on request prior to 39 weeks had changed to be 1.519(0.738–3.128). It implicated that the high possibility of total difficult problems in children with non-indicated ECS prior to 39 weeks was confounded by emotional problems. In other words, emotional problems might be the most important component in the overall emotional and behavioral abnormities in children born with ECS on maternal request prior to 39 gestational weeks. The increasing trend of preterm and early term deliveries is a growing and major public health concern [15, 44]. Previous studies indicated higher prevalence for inattention-hyperactivity, emotional problems and peer problems in preterm children [28, 29, 45, 46]. As far as conduct problems, results were much mixed and only a few studies found increased risk among very preterm children or children with very low birth weight [29, 47].

The mechanisms underlying the association of lower gestational age and higher emotional and behavior problems in children born with ECS on maternal request are complex and unclear. One possible mechanism might be the “iatrogenic prematurity” [12] that the pregnancy could be cut short when fetal brain development in the uterine is still going on [48]. Perinatal intervention like CS could interrupt the development of prefrontal cortex and hippocampal neurons in the experimental animals [49]. Hormonal secretion might be invoked to explain the association between ECS and children’s emotional and behavioral problems. The absence of labor will eliminate the release of stress hormones, such as glucocorticoid [50]. This process might have re-programming potentials on hypothalamic-pituitary-adrenal (HPA) axis function and behavior and result to long-term effects on children’s neuropsychological development [51, 52]. In this study, ECS with medical indications had shown a certain degree of protective effect on peer problems in children prior to 39 gestational weeks. Operative childbirth due to certain medical conditions was necessary intervention and could improve maternal and infant health outcomes. Its implication in protecting maternal and child health went beyond the process of labor, and the underlying beneficial neurodevelopmental effect on the children was quite different from non-indicated ECS.

The “39 week” rule for ECS on maternal request is mostly based on evidence of maternal and infant physiological effects in western populations. A potential psychological implication prior to 39 weeks in the present study has thus been added to the roster of impacts of ECS on maternal request. At various fetal maturity status, our conclusions have directed independent effect of ECS on request on children’s psychological outcomes and provided evidence-based data on maternal health providers’ and user’s decision-making of ECS and the optimal timing to perform it. The reasons for the very high overall CS rate and rate of ECS on maternal request are complex in China. Social/cultural factors, such as women’s fear of pain, selection of an auspicious birth day, bandwagon effect among maternal health users, as well as physician’s defensive medical behavior may play important major in the dramatic increase [53, 54]. Due to the limited development in labor analgesia and deep-rooted cultural contexts in China, it might be difficult to reverse the situation in a short period. As a concession, the findings have important implications for clinical practice in China, where there is no definite guideline on optimal timing of the non-indicated CS. The health education is not only limited among the providers. It is more critical to communicate to pregnant women and their families on the negative consequences of early ECS, because most women believed that full term was reaching 37 gestational weeks and it was safe to deliver at that time when there were no other complications [55]. Emotional and behavioral development should be closely monitored in children born with non-indicated ECS prior to 39 weeks in child health care service. Although the problems of early term are subtler than those of preterm babies, they are more numerous and thus possibly to be a greater burden for health services, and would benefit from more careful concern and assessment.

This study was a birth cohort design. The loss rate in the cohort was relatively low. It was around 4.2% from the 1st trimester of pregnancy to childbirth, and about 18.5% from delivery till 3–5 years after delivery. General characteristics of mother-infant pairs and children’s emotional and behavioral outcomes were collected prospectively so that recall bias was less likely to occur. This design also allowed for the inclusion of a large number of potential confounders. In particular, prenatal factors that might cause early elective childbirth and relate with offspring’s psychological development, such as prenatal stress and pregnant complications were fully considered and controlled. Differences in rate of prenatal anxiety were reported among different studies due to various population and instruments. The prevalence of pregnancy-related anxiety was 24.9 and 13.9% respectively in the 1st and 3rd trimester of pregnancy in our study. Other Chinese researchers reported maternal anxiety rate by using general anxiety scales. For example, with self-rating anxiety scale (SAS), 20.6% women had reported antenatal anxiety in late pregnancy [56]. Yu Y et al. revealed that 22.6 and 21.0% women had maternal anxiety respectively in 1st and 3rd trimester of pregnancy [57]. Pregnancy-related anxiety questionnaire developed by our team had been adopted by other researchers. 15.2 and 21.7% women were found to have maternal anxiety respectively in Shanghai [58] and Zhejiang [59], China.

Meanwhile, some weakness in the study must be acknowledged. Firstly, as to the participants, most of them were from urban areas. Maternal health service usage, breastfeeding situation and parenting style could be quite different between urban and rural families. Therefore, it should be prudent to explain our findings in rural areas. The overall prevalence of emotional and behavioral problems in pre-school children was reported to be 34.1 and 11.9% in rural areas respectively in Shandong and Hunan province, China [60, 61], being higher than the rate observed in our urban sample (7.6%). Secondly, in terms of the outcomes, although SDQ was reported to be a more methodologically robust method to assess emotional/behavioral problems among children [62] and was widely used in the clinical settings and community, it was a screening tool after all. It was difficult to use diagnostic instrument to confirm children’s neuropsychological status in the field with large sample size. Currently, researches specifically designed for predictive validity of SDQ in preschoolers on later childhood help seeking in China are limited. When normative data of the Chinese translation of the SDQ were described in a large group of children aged between 3 and 17 years old, researchers had performed validity analysis towards Chinese version of SDQ. It was proved to have favorable cross-scale correlation with the original UK description of the psychometric properties of the SDQ. Convergent validity was acceptable by analyzing between SDQ and PSQ (Conner’s Parent Symptom Questionnaire), and discriminant validity was verified when comparing respondents from the normative sample with ADHD outpatients matched for age and gender [34]. Predictive validity was testified among some school-aged children in China. It supported the ability of the Chinese SDQ to discriminate between community and clinic children [63, 64]. Thirdly, we were not able to rule out the potential for residual confounding. For example, parents who requested ECS, especially mothers, may be the same individuals that completed the SDQ. Therefore there might be a rater bias whereby variables that drove the request for ECS in the first place might also drive higher rates of SDQ problems and create an important confound to the current research that limited the ability to draw a link between CS and children’s emotional/behavioral problems. A total of 2455 mothers (74.0%) completed SDQ in our study, which might indicate that ECS on request and SDQ ratings were partly reflective of certain maternal characteristics. It may possibly represent an additional reason for elevated SDQ scores outside the effects of ECS on request. In addition, we didn’t have data on children’s cognitive development and environmental factors such as postnatal corticosteroids exposure and parenting style, as may mediate children’s emotional and social development [65–67]. There might be familial confounding that should be further considered. Based on a population-based sibling design study, Curran EA et al. [68, 69] had found the relationships between obstetric mode of delivery and autism spectrum disorder and attention-deficit/hyperactivity disorder, which were explained by familial confounding when sibling studies were performed.

## Conclusion

ECS on maternal request is found to be the independent predictor of emotional and behavioral problems in pre-school children born prior to 39 gestational weeks. The potential psychological implication has been added to the roster of impacts of ECS on maternal request before 39 weeks. It’s vital to communicate with both maternal health providers and users about the negative psychological consequences of early ECS in the absence of medical indications. Further research is needed to probe the potential biological mechanisms.

## Additional files

Additional file 1: Questionnaire of maternal and children’s health in China-Anhui Birth Cohort Study (DOCX 25 kb)

Additional file 2: **Table S1.** Comparison of basic maternal characteristics between women who were recruited in data analysis and those who were dropped out. **Table S2.** Binary logistic regression analysis on effect of delivery mode on SDQ in children born with various gestational ages (restricted to boys). **Table S3.** Binary logistic regression analysis on effect of delivery mode on SDQ in children born with various gestational ages (restricted to women without pregnancy-related anxiety). **Table S4.** Binary logistic regression analysis on effect of delivery mode on SDQ in children born with various gestational ages (restricted to women without pregnant complications). **Table S5.** Binary logistic regression analysis on effect of delivery mode on SDQ in children born with various gestational ages (restricted to women with normal pre-pregnancy BMI). **Table S6.** Binary logistic regression analysis on effect of delivery mode on SDQ in children born with various gestational ages (emergency caesarean section as the reference) (DOC 192 kb)

## Abbreviations

BMI: Body mass index
C-ABCS: China-Anhui Birth Cohort Study
ECS: Elective caesarean section
HPA axis: Hypothalamic-pituitary-adrenal axis
NICU: Neonatal intensive care unit
SDQ: Strengths and Difficulties Questionnaires
SEN: Special education needs
WHOGS: WHO Global Survey of Maternal and Perinatal Health
WHOMCS: WHO Multi-Country Survey of Maternal and Newborn Health
