# Supplementary material for: Elective caesarean section on maternal request prior to 39 gestational weeks and childhood psychopathology: a birth cohort study in China
Source: BMC Psychiatry. 2019 Jan 14;19:22. doi: 10.1186/s12888-019-2012-z (PMC6332907; doi:10.1186/s12888-019-2012-z)
Supplement: Supplementary file 1 — Questionnaire of maternal and children’s health in China-Anhui Birth Cohort Study (DOCX 25 kb) [file 12888_2019_2012_MOESM1_ESM.docx]

**Questionnaire of maternal and children’s health in China-Anhui Birth Cohort Study**

**(main variables used in the current study)**

**Contacting information**

1. Number of questionnaire:

2. Name of the pregnant women:

3. Maternal ID card number:

4. Contact information: Cell phone Email address:

5. Living address: province city county/community street/township village

province city community street district building room

6. Date for questionnaire survey: YYYY/MM/DD

**Maternal demographic characteristics**

1. Mother’s birthday : YYYY/MM/DD

2. Current living place:

(1) Rural areas (2) Urban areas

3. Total educational years: years

4. Monthly family income per capita:

(1) ≤2000yuan (2)2001-7999yuan (5)≥8000yuan

**History of pregnancy and childbirth**

1. Did you use assisted reproductive technology for the index pregnancy?

(1) yes (2) no

2. Did you experience the following pregnancy outcomes? (please fill in numbers if you had any of the outcomes and fill in 0 if you didn’t have):

spontaneous abortion induced abortion

vacuum/forceps use during vaginal delivery cystic mole ectopic pregnancy

newborn death fetal death/stillbirth caesarean section

delivered preterm baby (≥28 and <37 gestational weeks)

delivered full-term baby (≥37 and <42 gestational weeks)

delivered babies with congenital malformations others

**Pregnancy-related anxiety questionnaire**

| **Items** | **Never** | **Occasionally** | **Often** | **Always** |
| --- | --- | --- | --- | --- |
| 1. Do you worry about the opposite baby’s gender as the family expected? |  |  |  |  |
| 2. Do you worry that you don’t prepare enough for the index pregnancy? |  |  |  |  |
| 3. Do you worry that pregnancy will affect your job? |  |  |  |  |
| 4. Do you worry that pregnancy and childbirth will change your body shape? |  |  |  |  |
| 5. Do you worry that pregnancy will reduce your attractiveness to your husband? |  |  |  |  |
| 6. Do you worry that pregnancy will cause family’s economic burden? |  |  |  |  |
| 7. Do you worry that the baby is unhealthy (eg. malformation)? |  |  |  |  |
| 8. Do you worry that the baby has intelligence disability? |  |  |  |  |
| 9. Do you worry that your dietary pattern will affect the baby’s health? |  |  |  |  |
| 10. Do you worry that your sickness (eg. influenza, fever) will affect baby’s health? |  |  |  |  |
| 11. Do you worry about the pain of childbirth? |  |  |  |  |
| 12. Do you worry that you will have difficult labor during childbirth? |  |  |  |  |
| 13. Do you worry that your previous unhealthy life pattern will cause some adverse pregnancy outcome? |  |  |  |  |

**Information of preschool health and behaviors:**

1. Number of questionnaire:

2. Name of the mother:

3. Maternal ID card number:

4. Date for questionnaire survey: YYYY/MM/DD

5. Who fill in the questionnaire: (1) father (2) mother (3) grand-parents (4) others

6. Children’s birth date: YYYY/MM/DD

7. Number of fetus: (1) singleton (2) twins (3) triplet

8. Feeding pattern in 4 months:

(1) exclusive breastfeeding; (2) almost exclusive breastfeeding (providing additional water or juice); (3) high partial breastfeeding (breastfeeding accounting for over 80% of total baby’ s food); (4) medium partial breastfeeding (breastfeeding accounting for 20-79% of total baby’s food); (5) low partial breastfeeding (breastfeeding accounting for less than 20% of total baby’s food); (6) token breastfeeding (breastfeeding just for comforting baby, not for providing calories); (7) artificial feeding (no breastfeeding).

9. Feeding pattern in 6 months:

(1) exclusive breastfeeding; (2) almost exclusive breastfeeding (providing additional water or juice); (3) high partial breastfeeding (breastfeeding accounting for over 80% of total baby’ s food); (4) medium partial breastfeeding (breastfeeding accounting for 20-79% of total baby’s food); (5) low partial breastfeeding (breastfeeding accounting for less than 20% of total baby’s food); (6) token breastfeeding (breastfeeding just for comforting baby, not for providing calories); (7) artificial feeding (no breastfeeding).

10. SDQ

| Items | not true | somewhat true | certainly true |
| --- | --- | --- | --- |
| 1. Considerate of other people’s feelings |  |  |  |
| 2. Restless, overactive, cannot stay still for long |  |  |  |
| 3. Often complains of headaches, stomach-aches or sickness |  |  |  |
| 4. Shares readily with other children (treats, toys, pencils etc.) |  |  |  |
| 5. Often has temper tantrums or hot tempers |  |  |  |
| 6. Rather solitary, tends to play alone |  |  |  |
| 7. Generally obedient, usually does what adults request |  |  |  |
| 8. Many worries, often seems worried |  |  |  |
| 9. Helpful if someone is hurt, upset or feeling ill |  |  |  |
| 10. Constantly fidgeting or squirming |  |  |  |
| 11. Has at least one good friend |  |  |  |
| 12. Often fights with other children or bullies them |  |  |  |
| 13. Often unhappy, down-hearted or tearful |  |  |  |
| 14. Generally liked by other children |  |  |  |
| 15. Easily distracted, concentration wanders |  |  |  |
| 16. Nervous or clingy in new situations, easily loses confidence |  |  |  |
| 17. Kind to younger children |  |  |  |
| 18. Often lies or cheats |  |  |  |
| 19. Picked on or bullied by other children |  |  |  |
| 20. Often volunteers to help others |  |  |  |
| 21. Thinks things out before acting |  |  |  |
| 22. Steals from home, school or elsewhere |  |  |  |
| 23. Gets on better with adults than with other children |  |  |  |
| 24. Many fears, easily scared |  |  |  |
| 25. Sees tasks through to the end, good attention span |  |  |  |
