# Supplementary material for: Elective caesarean section on maternal request prior to 39 gestational weeks and childhood psychopathology: a birth cohort study in China
Source: BMC Psychiatry. 2019 Jan 14;19:22. doi: 10.1186/s12888-019-2012-z (PMC6332907; doi:10.1186/s12888-019-2012-z)
Supplement: Supplementary file 2 — Table S1. Comparison of basic maternal characteristics between women who were recruited in data analysis and those who were dropped out. Table S2. Binary logistic regression analysis on effect of delivery mode on SDQ in children born with various gestational ages (restricted to boys). Table S3. Binary logistic regression analysis on effect of delivery mode on SDQ in children born with various gestational ages (restricted to women without pregnancy-related anxiety). Table S4. Binary logistic regression analysis on effect of delivery mode on SDQ in children born with various gestational ages (restricted to women without pregnant complications). Table S5. Binary logistic regression analysis on effect of delivery mode on SDQ in children born with various gestational ages (restricted to women with normal pre-pregnancy BMI). Table S6. Binary logistic regression analysis on effect of delivery mode on SDQ in children born with various gestational ages (emergency caesarean section as the reference) (DOC 192 kb) [file 12888_2019_2012_MOESM2_ESM.doc]

**Table S1 Comparison of basic maternal characteristics between women who were recruited in data analysis and those who were dropped out**

| **Characteristics** | **Women who were recruited in data analysis (n=3319)** | **Women who were dropped out (n=1770)** |
| --- | --- | --- |
| Maternal age (years) (mean±SD) | 26.7±3.6 | 26.8±3.6 |
| Maternal educational years (mean±SD) | 12.8±3.3 | 12.8±3.5 |
| Residence (n/%) ^a^** |  |  |
| Urban areas | 2765/83.4 | 1548/87.6 |
| Rural areas | 549/16.6 | 219/12.4 |
| Family monthly income per capita (n/%) ^b^** |  |  |
| ≤2000 RMB yuan | 1926/58.1 | 1027/58.1 |
| 2001-7999 RMB yuan | 1341/40.5 | 726/41.1 |
| ≥8000 RMB yuan | 46/1.4 | 14/0.8 |
| Previous adverse pregnant outcomes (n/%) | 1613/48.6 | 853/48.2 |
| Pre-pregnancy BMI (kg/m^2^) (mean±SD) | 20.2±2.4 | 20.3±2.5 |
| Primiparity (n/%) | 3122/94.1 | 1667/94.2 |
| Pregnancy-related anxiety scores in 1^st^ trimester of pregnancy (mean±SD) | 21.1±5.2 | 21.0±5.1 |
| Pregnancy-related anxiety in 1^st^ trimester of pregnancy (n/%) | 870/26.2 | 448/25.3 |

* P＜0.05, ** P＜0.01

^a^ 5 missing values in women who were recruited in data analysis and 3 missing values in women who were dropped out

^b^ 6 missing values in women who were recruited in data analysis and 3 missing values in women who were dropped out

**Table S2 Binary logistic regression analysis on effect of delivery mode on SDQ in children born with various gestational ages [RR (95%CI)]**

(restricted to boys)

| **SDQ dimensions** | **Mode of delivery** | **<39 weeks** | **39-40 weeks** | **≥41 weeks** | **Total** |
| --- | --- | --- | --- | --- | --- |
| Emotional problems | ECS with medical indications | 2.225(0.757-6.537) | 1.375(0.737-2.565) | 2.091(0.178-24.500) | 1.137(0.679-1.902) |
|  | ECS on maternal request | 6.796(2.283-20.225) | 0.933(0.440-1.978) | NA | 1.065(0.579-1.958) |
|  | Emergency caesarean section | 1.113(0.122-10.187) | 0.743(0.244-2.261) | 3.047(0.253-36.693) | 0.717(0.292-1.764) |
| Conduct problems | ECS with medical indications | 0.657(0.303-1.426) | 0.890(0.502-1.580) | 2.295(0.550-9.582) | 0.933(0.608-1.434) |
|  | ECS on maternal request | 1.414(0.599-3.335) | 1.435(0.812-2.534) | 0.636(0.061-6.655) | 1.415(0.897-2.235) |
|  | Emergency caesarean section | 0.444(0.096-2.061) | 0.783(0.312-1.966) | 1.035(0.187-5.715) | 0.756(0.381-1.499) |
| Hyperactivity | ECS with medical indications | 1.093(0.559-2.137) | 0.550(0.300-1.011) | 0.224(0.064-0.184) | 0.704(0.471-1.054) |
|  | ECS on maternal request | 1.029(0.417-2.540) | 1.010(0.569-1.793) | 0.143(0.017-1.201) | 0.849(0.539-1.338) |
|  | Emergency caesarean section | 0.437(0.096-1.994) | 0.433(0.148-1.269) | 0.479(0.146-1.573) | 0.571(0.292-1.118) |
| Peer problems | ECS with medical indications | 0.290(0.088-0.954) | 0.920(0.437-1.938) | NA | 0.701(0.382-1.288) |
|  | ECS on maternal request | 0.473(0.100-2.233) | 1.123(0.515-2.448) | NA | 0.971(0.497-1.896) |
|  | Emergency caesarean section | 1.509(0.389-5.851) | 0.480(0.108-2.141) | NA | 0.987(0.419-2.327) |
| Total difficult problems | ECS with medical indications | 1.410(0.626-3.172) | 0.838(0.457-1.534) | 0.541(0.107-2.719) | 1.065(0.679-1.672) |
|  | ECS on maternal request | 3.378(1.399-8.152) | 1.534(0.858-2.741) | NA | 1.824(1.142-2.913) |
|  | Emergency caesarean section | 0.813(0.170-3.883) | 0.756(0.299-1.912) | 1.066(0.233-4.881) | 0.983(0.502-1.927) |
| Pro-social behaviors | ECS with medical indications | 0.960(0.530-1.739) | 0.841(0.546-1.296) | 0.841(0.546-1.296) | 0.921(0.661-1.284) |
|  | ECS on maternal request | 0.958(0.431-2.129) | 1.195(0.784-1.867) | 1.195(0.764-1.867) | 1.191(0.821-1.727) |
|  | Emergency caesarean section | 0.679(0.221-2.092) | 0.549(0.251-1.199) | 0.549(0.251-1.199) | 0.619(0.352-1.090) |

Vaginal delivery as the reference group.

Adjusted for maternal age, maternal education level, residence, family income, previous adverse pregnant outcomes, pre-pregnancy BMI, pregnant complications, parity, pregnancy-related anxiety, birth weight and 5min Apgar scores

**Table S3 Binary logistic regression analysis on effect of delivery mode on SDQ in children born with various gestational ages [RR (95%CI)]**

**(restricted to women without pregnancy-related anxiety**)

| **SDQ dimensions** | **Mode of delivery** | **<39 weeks** | **39-40 weeks** | **≥41 weeks** | **Total** |
| --- | --- | --- | --- | --- | --- |
| Emotional problems | ECS with medical indications | 1.114(0.511-2.430) | 1.451(0.807-2.607) | 1.212(0.292-5.033) | 1.198(0.755-1.898) |
|  | ECS on maternal request | 2.506(1.115-5.635) | 0.905(0.442-1.854) | NA | 1.106(0.642-1.907) |
|  | Emergency caesarean section | 0.457(0.057-3.644) | 0.841(0.282-2.507) | 1.001(0.176-5.693) | 0.749(0.328-1.711) |
| Conduct problems | ECS with medical indications | 0.674(0.344-1.320) | 1.075(0.813-1.887) | 2.196(0.754-6.401) | 0.959(0.641-1.435) |
|  | ECS on maternal request | 1.837(0.924-3.655) | 1.160(0.638-2.109) | 0.935(0.218-4.010) | 1.200(0.769-1.873) |
|  | Emergency caesarean section | 0.790(0.225-2.778) | 0.790(0.295-2.115) | 0.549(0.104-2.891) | 0.705(0.352-1.411) |
| Hyperactivity | ECS with medical indications | 0.703(0.383-1.293) | 1.051(0.594-1.858) | 0.510(0.168-1.547) | 0.840(0.559-1.264) |
|  | ECS on maternal request | 0.836(0.384-1.821) | 0.818(0.414-1.617) | 0.324(0.068-1.532) | 0.630(0.373-1.064) |
|  | Emergency caesarean section | 0.429(0.098-1.888) | 0.303(0.071-1.298) | 0.699(0.208-2.342) | 0.516(0.242-1.100) |
| Peer problems | ECS with medical indications | 0.275(0.094-0.802) | 0.843(0.359-1.981) | 0.391(0.040-3.848) | 0.530(0.277-1.014) |
|  | ECS on maternal request | 0.661(0.207-2.108) | 0.889(0.343-2.303) | NA | 0.810(0.282-1.317) |
|  | Emergency caesarean section | 0.904(0.194-4.211) | 0.619(0.135-2.625) | 0.659(0.066-6.530) | 0.729(0.276-1.930) |
| Total difficult problems | ECS with medical indications | 0.926(0.487-1.762) | 1.122(0.605-2.080) | 0.958(0.247-3.712) | 1.0099(0.643-1.581) |
|  | ECS on maternal request | 2.031(1.019-4.046) | 1.593(0.841-3.017) | 0.376(0.043-3.326) | 1.434(1.084-2.324) |
|  | Emergency caesarean section | 0.561(0.126-2.505) | 1.105(0.435-2.806) | 1.663(0.422-6.549) | 1.124(0.579-2.182) |
| Pro-social behaviors | ECS with medical indications | 1.147(0.656-2.005) | 0.868(0.573-1.315) | 1.238(0.400-3.831) | 0.962(0.695-1.332) |
|  | ECS on maternal request | 1.627(0.822-3.223) | 1.132(0.730-1.754) | 1.701(0.536-5.403) | 1.250(0.872-1.793) |
|  | Emergency caesarean section | 1.099(0.395-3.058) | 0.676(0.334-1.371) | 0.624(0.118-3.313) | 0.687(0.392-1.202) |

Vaginal delivery as the reference group.

NA: not applicable.

Adjusted for maternal age, maternal education level, residence, family income, previous adverse pregnant outcomes, pre-pregnancy BMI, pregnant complications, parity, children’ gender, birth weight and 5min Apgar scores

**Table S4 Binary logistic regression analysis on effect of delivery mode on SDQ in children born with various gestational ages [RR (95%CI)]**

**(restricted to women without pregnant complications**)

| **SDQ dimensions** | **Mode of delivery** | **<39 weeks** | **39-40 weeks** | **≥41 weeks** | **Total** |
| --- | --- | --- | --- | --- | --- |
| Emotional problems | ECS with medical indications | 1.018(0.459-2.261) | 1.148(0.717-1.839) | 1.144(0.278-4.709) | 1.133(0.767-1.674) |
|  | ECS on maternal request | 2.954(1.346-6.480) | 0.800(0.453-1.412) | 0.505(0.055-4.634) | 1.200(0.777-1.852) |
|  | Emergency caesarean section | 0.433(0.055-3.430) | 1.068(0.519-2.198) | 2.809(0.760-10.378) | 1.206(0.685-2.123) |
| Conduct problems | ECS with medical indications | 0.596(0.306-1.158) | 0.918(0.574-1.469) | 1.627(0.589-4.492) | 0.878(0.619-1.247) |
|  | ECS on maternal request | 1.789(0.930-3.441) | 1.332(0.837-2.122) | 0.774(0.184-3.251) | 1.369(0.953-1.966) |
|  | Emergency caesarean section | 0.413(0.094-1.813) | 0.614(0.270-1.394) | 0.978(0.295-3.242) | 0.690(0.389-1.223) |
| Hyperactivity | ECS with medical indications | 0.660(0.358-1.215) | 1.101(0.682-1.778) | 0.453(0.167-1.231) | 0.869(0.504-1.498) |
|  | ECS on maternal request | 1.181(0.595-2.343) | 1.248(0.781-2.047) | 0.554(0.173-1.774) | 1.165(0.646-2.101) |
|  | Emergency caesarean section | 0.361(0.082-1.588) | 0.419(0.147-1.193) | 0.861(0.327-2.265) | 1.131(0.528-2.424) |
| Peer problems | ECS with medical indications | 0.339(0.115-1.001) | 1.317(0.656-2.645) | 0.754(0.123-4.610) | 0.701(0.382-1.288) |
|  | ECS on maternal request | 0.739(0.229-2.380) | 1.506(0.717-3.161) | 0.680(0.069-6.698) | 0.971(0.497-1.896) |
|  | Emergency caesarean section | 1.412(0.376-5.296) | 1.047(0.342-3.208) | 1.175(0.191-7.235) | 0.987(0.419-2.327) |
| Total difficult problems | ECS with medical indications | 1.033(0.547-1.948) | 1.038(0.646-1.667) | 0.749(0.225-2.500) | 1.019(0.711-1.461) |
|  | ECS on maternal request | 2.409(1.230-4.719) | 1.619(0.013-2.588) | 0.600(0.120-3.012) | 1.681(1.162-2.431) |
|  | Emergency caesarean section | 0.505(0.114-2.243) | 1.075(0.538-2.148) | 2.265(0.772-6.640) | 1.160(0.696-1.934) |
| Pro-social behaviors | ECS with medical indications | 1.025(0.580-1.813) | 0.759(0.523-1.101) | 1.019((0.378-2.750) | 0.858(0.639-1.153) |
|  | ECS on maternal request | 1.524(0.781-2.973) | 1.002(0.680-1.477) | 1.245(0.407-3.810) | 1.183(0.858-1.631) |
|  | Emergency caesarean section | 0.640(0.210-1.945) | 0.748(0.417-1.347) | 0.713(0.217-2.339) | 0.737(0.462-1.175) |

Vaginal delivery as the reference group.

Adjusted for maternal age, maternal education level, residence, family income, previous adverse pregnant outcomes, pre-pregnancy BMI, parity, pregnancy-related anxiety, children’ gender, birth weight and 5min Apgar scores

**Table S5 Binary logistic regression analysis on effect of delivery mode on SDQ in children born with various gestational ages [RR (95%CI)]**

**(restricted to women with normal pre-pregnancy BMI**)

| **SDQ dimensions** | **Mode of delivery** | **<39 weeks** | **39-40 weeks** | **≥41 weeks** | **Total** |
| --- | --- | --- | --- | --- | --- |
| Emotional problems | ECS with medical indications | 1.126(0.532-2.380) | 1.083(0.693-1.694) | 0.776(0.176-3.418) | 1.024(0.709-1.480) |
|  | ECS on maternal request | 3.086(1.445-6.589) | 0.949(0.575-1.569) | 0.388(0.043-3.467) | 1.239(0.833-1.844) |
|  | Emergency caesarean section | 0.357(0.046-2.797) | 0.893(0.440-1.815) | 2.691(0.789-9.180) | 1.051(0.611-1.807) |
| Conduct problems | ECS with medical indications | 0.725(0.392-1.340) | 0.878(0.562-1.369) | 1.370(0.532-3.530) | 0.912(0.655-1.271) |
|  | ECS on maternal request | 1.720(0.900-3.289) | 1.403(0.915-2.153) | 0.363(0.073-1.801) | 1.361(0.966-1.918) |
|  | Emergency caesarean section | 0.520(0.150-1.799) | 0.611(0.284-1.316) | 0.670(0.204-2.201) | 0.674(0.388-1.169) |
| Hyperactivity | ECS with medical indications | 0.709(0.403-1.247) | 0.985(0.622-1.559) | 0.231(0.074-0.723) | 0.844(0.603-1.193) |
|  | ECS on maternal request | 0.964(0.485-1.913) | 1.308(0.831-2.059) | 0.420(0.132-1.338) | 1.146(0.802-1.637) |
|  | Emergency caesarean section | 0.544(0.182-1.626) | 0.433(0.169-1.110) | 0.635(0.244-1.653) | 0.693(0.398-1.207) |
| Peer problems | ECS with medical indications | 0.212(0.067-0.669) | 1.013(0.501-2.052) | 0.389(0.040-3.804) | 0.613(0.349-1.077) |
|  | ECS on maternal request | 0.639(0.203-2.017) | 1.448(0.723-2.903) | 0.648(1.066-6.380) | 1.097(0.628-1.917) |
|  | Emergency caesarean section | 1.147(0.313-4.209) | 0.935(0.310-2.822) | 0.565(0.057-5.546) | 0.886(0.404-1.943) |
| Total difficult problems | ECS with medical indications | 0.922(0.502-1.694) | 1.068(0.680-1.678) | 0.464(0.134-1.810) | 0.999(0.707-1.412) |
|  | ECS on maternal request | 2.012(1.048-3.861) | 1.621(0.049-2.504) | 0.435(0.090-2.094) | 1.649(1.164-2.338) |
|  | Emergency caesarean section | 0.570(0.164-1.988) | 0.899(0.443-1.825) | 1.439(0.505-4.102) | 0.982(0.589-1.639) |
| Pro-social behaviors | ECS with medical indications | 1.106(0.654-1.869) | 0.921(0.644-1.317) | 1.285(0.473-3.486) | 0.979(0.739-1.296) |
|  | ECS on maternal request | 1.388(0.727-2.650) | 1.137(0.788-1.641) | 1.229(0.401-3.784) | 1.226(0.902-1.664) |
|  | Emergency caesarean section | 0.764(0.282-2.074) | 0.756(0.422-1.354) | 1.032(0.332-3.209) | 0.793(0.507-1.242) |

Vaginal delivery as the reference group.

Adjusted for maternal age, maternal education level, residence, family income, previous adverse pregnant outcomes, pregnant complications, parity, pregnancy-related anxiety, children’ gender, birth weight and 5min Apgar scores

**Table S6 Binary logistic regression analysis on effect of delivery mode on SDQ in children born with various gestational ages [RR (95%CI)]**

**(emergency caesarean section as the reference)**

| **SDQ dimensions** | **Mode of delivery** | **<39 weeks** | **39-40 weeks** | **≥41 weeks** | **Total** |
| --- | --- | --- | --- | --- | --- |
| Emotional problems | Vaginal delivery | 2.681(0.343-20.968) | 1.017(0.511-2.024) | 0.401(0.130-1.240) | 0.865(0.513-1.460) |
|  | ECS with medical indications | 3.414(0.448-25.999) | 1.118(0.560-2.232) | 0.366(0.112-1.194) | 0.957(0.569-1.609) |
|  | ECS on maternal request | 9.326(1.220-71.291) | 0.855(0.409-1.788) | 0.134(0.016-1.128) | 1.081(0.626-1.867) |
| Conduct problems | Vaginal delivery | 1.776(0.514-6.135) | 1.613(0.748-3.480) | 1.025(0.348-3.020) | 1.326(0.783-2.245) |
|  | ECS with medical indications | 1.309(0.378-4.534) | 1.360(0.617-2.997) | 1.389(0.511-3.777) | 1.143(0.669-1.950) |
|  | ECS on maternal request | 3.102(0.882-10.911) | 2.107(0.965-4.602) | 0.628(0.149-2.648) | 1.764(1.027-3.032) |
| Hyperactivity | Vaginal delivery | 1.757(0.590-5.235) | 2.344(0.913-6.019) | 1.457(0.584-3.640) | 1.575(0.919-2.697) |
|  | ECS with medical indications | 1.391(0.469-4.123) | 2.249(0.866-5.840) | 0.456(0.150-1.388) | 1.292(0.747-2.235) |
|  | ECS on maternal request | 1.685(0.531-5.344) | 2.712(0.042-7.061) | 0.575(0.162-2.035) | 1.545(0.877-2.721) |
| Peer problems | Vaginal delivery | 0.920(0.254-3.331) | 1.085(0.359-3.284) | 0.882(0.144-5.411) | 1.037(0.491-2.192) |
|  | ECS with medical indications | 0.227(0.052-0.997) | 1.204(0.400-3.625) | 0.638(0.088-4.639) | 0.721(0.332-1.568) |
|  | ECS on maternal request | 0.649(0.149-2.827) | 1.466(0.481-4.472) | 0.609(0.054-6.897) | 1.103(0.500-2.432) |
| Total difficult problems | Vaginal delivery | 1.718(0.496-5.944) | 1.048(0.527-2.085) | 0.508(0.194-1.331) | 0.883(0.545-1.429) |
|  | ECS with medical indications | 1.704(0.500-5.811) | 1.049(0.519-2.120) | 0.310(0.105-1.914) | 0.884(0.544-1.438) |
|  | ECS on maternal request | 3.687(1.059-12.835) | 1.596(0.794-3.206) | 0.255(0.053-1.230) | 1.374(0.839-2.249) |
| Pro-social behaviors | Vaginal delivery | 1.407(0.520-3.806) | 1.320(0.750-2.324) | 1.097(0.364-3.304) | 1.287(0.829-1.996) |
|  | ECS with medical indications | 1.488(0.558-3.971) | 1.101(0.618-1.963) | 1.126(0.359-3.536) | 1.157(0.743-1.802) |
|  | ECS on maternal request | 1.717(0.599-4.922) | 1.494(0.835-2.674) | 1.586(0.467-5.389) | 1.566(0.990-2.477) |

Emergency caesarean section as the reference group.

Adjusted for maternal age, maternal education level, residence, family income, previous adverse pregnant outcomes, pre-pregnancy BMI, pregnant complications, parity, pregnancy-related anxiety, children’ gender, birth weight and 5min Apgar scores
